# The Impact of Elongation on Change in Electrical Resistance of Electrically Conductive Yarns Woven into Fabric

**DOI:** 10.3390/ma14123390

**Published:** 2021-06-18

**Authors:** Željko Knezić, Željko Penava, Diana Šimić Penava, Dubravko Rogale

**Affiliations:** 1 Department of Textile Design and Management, Faculty of Textile Technology, University of Zagreb, 10000 Zagreb, Croatia; zeljko.knezic@ttf.unizg.hr (Ž.K.); zpenava@ttf.unizg.hr (Ž.P.); 2 Department of Engineering Mechanics, Faculty of Civil Engineering, University of Zagreb, 10000 Zagreb, Croatia; dianas@grad.unizg.hr; 3 Department of Clothing Technology, Faculty of Textile Technology, University of Zagreb, 10000 Zagreb, Croatia

**Keywords:** woven fabric, plain weave, electrically conductive yarn, resistance, tensile force, elongation

## Abstract

Electrically conductive yarns (ECYs) are gaining increasing applications in woven textile materials, especially in woven sensors suitable for incorporation into clothing. In this paper, the effect of the yarn count of ECYs woven into fabric on values of electrical resistance is analyzed. We also observe how the direction of action of elongation force, considering the position of the woven ECY, effects the change in the electrical resistance of the electrically conductive fabric. The measurements were performed on nine different samples of fabric in a plain weave, into which were woven ECYs with three different yarn counts and three different directions. Relationship curves between values of elongation forces and elongation to break, as well as relationship curves between values of electrical resistance of fabrics with ECYs and elongation, were experimentally obtained. An analytical mathematical model was also established, and analysis was conducted, which determined the models of function of connection between force and elongation, and between electrical resistance and elongation. The connection between the measurement results and the mathematical model was confirmed. The connection between the mathematical model and the experimental results enables the design of ECY properties in woven materials, especially textile force and elongation sensors.

## 1. Introduction

Electrically conductive textiles represent a new generation of textile fibers, yarns, fabrics, and knits, with a wide range of uses [1]. Mattila therefore expands the field of conventional textiles, regarding their integrative complexity and their degree of integration. Conventional textiles retain low complexity and a low degree of integration. So-called functional textiles retain low complexity but take on a high degree of integration. Wearable electronics are attached to but not integrated into textile structures. When electronics and other devices are integrated directly into fibers and textiles, it is called textronics and fibertronics, and represents the highest degree of integration and complexity [2]. Therefore, electrically conductive textiles play an increasingly important role in the production of intelligent clothing, surveillance and protective clothing, energy-producing clothing, clothing for medical diagnostics, for heating, etc., and are used in the production of uniforms for the army, police, firefighters, and other special services. Gao et al. [3] note that highly stretchable sensors for wearable biomedical applications have attracted substantial attention since their appearance in the early twenty-first century, due to their unique characteristics, such as low modulus, light weight, high flexibility, and stretchability.

According to the working environment, Cheng et al. [4] divided flexible force sensors into wearable and implantable sensors. Wearable sensors are still limited by their complex structure, difficult material handling, and dependence on external power supply.

Nascimento et al. [5] consider the importance of sensors for the monitoring and protection of human health, as well as methods applied for physical and motor rehabilitation, which they predict will be linked to machine learning, artificial intelligence, and the Internet of things (IoT) in the near future.

Liu et al. [6] made an overview of the development of indicators and sensors for health monitoring, and divided them into five groups: body motion, skin temperature, heart rate/ECG/pulse, metabolism, and respiration. Measurement parameters for the respiration indicator are strain, pressure, and humidity for detection of cardiac arrest, apnea, and emotional control. In particular, wearable sensors installed on the chest/abdomen can be used to detect respiration.

Ehrmann et al. [7] grouped the possible applications of ECYs for electromagnetic shielding, textile pressure sensors, antielectrostatic textiles, and electronic circuits in plain textile materials, as well as their most common use in elongation sensors.

Such textile materials consist of electrically conductive fibers and yarn and thread, irrespective of whether such electrically conductive yarn is only partially incorporated or the whole flat textile product is made of it. In ECY, in addition to the electrical conductivity of the fibers, the total conductivity will also depend on the average length of the fiber within the thread, the formation of permanent joints, and the electrical resistance present at the contact areas with adjacent fibers [8]. It is known that twisting yarn compresses the fibers as well as shearing, joining, and arranging them into a more compact structure. It is to be expected that this creates additional and alternative electrically conductive contacts that directly affect the overall electrical conductivity of the yarn [9]. Accordingly, it can be assumed that similar or identical behavior of ECY used for textile sensors increases the number of shares or twists required to improve the linearity such that the sensitivity could achieve the minimum requirements [10].

Some studies have described the design and development of a flexible sensor for measuring textile deformations based on a conductive polymer composite [11].

In electrically conductive textiles, the length-associated electrical resistance and the contact resistance contribute the most to the overall resistance [12]. The length-related resistance depends only on the length of the electrically conductive yarn when the tensile force acts in parallel with the electrically conductive yarn, while the contact resistance depends on the contact surface under the tensile force [13,14,15]. Ryu et al. provided a theoretical analysis showing the effect of changes in the deformation of electrically conductive yarn on electrical resistance [16]. From theoretical analysis and experimental research, it was determined that the contact resistance of folded yarns in the fabric is a key factor affecting the sensitivity of the sensor embedded in the fabric [14]. The application of sensors in the conductive fabric is based on changes in electrical resistance that respond to stimuli such as deformation, temperature, and humidity [17]. Several papers have described experimental studies of the relationship between load/elongation and electrical resistance of electrically conductive fabrics [18,19].

Roh [20], Tangsirinaruenart and Stylios [21], and Ruppert-Stroescu and Balasubramanian [22] successfully fastened ECY to textile materials by sewing or embroidery using different types of machine sewing stitches, obtaining similar results of changes in resistance with elongation as in the case of weaving and knitting. Much of the research on textile strain sensors is based on the use of metal wires or electrically conductive yarns from well-known manufacturers. Eom et al. [23] show that it is possible to treat textiles with their own chemical substances and then sew them to the textile materials with flat and zigzag sewing stitches.

Ivšić et al. [24] show that with the embroidery technique it is possible to make wearable antennas on clothes with the help of conductive threads for high frequencies. Garnier et al. show that antennas on clothing at a short-wave frequency can also be obtained using the embroidery technique [25]. Chang et al. [26] dedicated themselves to the development of stretchable microwave antenna systems, considering that wireless functionality is essential for the implementation of wearable systems. They replaced the textile substrate with elastomeric substrates, and obtained better results due to changes in the dimensions of the antenna elements.

It is evident that ECYs have a good potential for future application. Thus, Yang et al. see the use of conductive yarns as flexible actuators for soft robotics [27], while Kan and Lam foresee a future trend in wearable electronics in the textile industry [28].

Yao and Zhu [29] state that the development of wearable multifunctional sensors assumes the extent to which woven or textile materials are replaced by printed, stretchable conductors made of silver nanowires with excellent properties.

Ha et al. [30] have researched wearable and flexible sensors based on engineered functional nano/micro-materials, with unique sensing capabilities for detection of the physical and electrophysiological vital signs of humans. One easy way to make stretchable conductors is the use of conductive nanomaterials—such as graphene, conducting polymers, and liquid metals—in combination with an intrinsically stretchable elastomeric polymer matrix.

However, some authors have already discovered some defects of the electrically conductive filaments, so this will need to be considered in future research. Stavrakis et al. indicate the peeling of silver-plated layers [31] when too much electric current passes through the yarn. Another problem is cyclic loading, at which unwanted hysteresis occurs, such that the data are not repeatable after a certain number of load cycles; this is indicated by McKnight et al. [32]. A similar problem was noted by Wang et al., who examined intrinsically stretchable and conductive textiles by a scalable process for elastic wearable electronics [33]. The durability of the conductive threads used for integration of electronics into smart clothing, over several washing cycles of clothing, was examined by Briedis et al., who found that the values of the parameters of the ECYs decrease with more washing cycles [34].

The research presented in this paper is focused on determining the impact of elongation on the change in electrical resistance of the ECYs woven into fabric, and later on the development of motion sensors that change electrical resistance due to elongation.

ECY was used in a traditionally woven ribbon with variable electrical resistance when voltage is changed. Textile ribbon with ECY could be used in the garment as a motion sensor to monitor the breathing of people with apnea, and this innovation was recognized and awarded at international exhibitions by two special awards, five gold medals, and one silver medal. These awards show the innovation of the traditionally woven ribbon, and the applicability of electrically conductive yarn. This innovation was used in the breathing simulator, i.e. chest movement simulator.

The aim of this paper is to investigate how the change in the elongation of the samples under the action of tensile force affects the change in the electrical resistance of ECY woven into fabrics.

## 2. Materials and Methods

### 2.1. Measuring the Electrical Resistance

The ohmic resistance of a conductor (or of a resistor) can be measured by several instruments and by various methods, e.g., by measuring current and voltage with ammeters and voltmeters; by comparing the known with unknown resistance; or by using ordinary and digital ohmmeters, as well as different measuring bridges. For this research, the so-called comparative method of resistance measurement is used.

**Figure 1:**
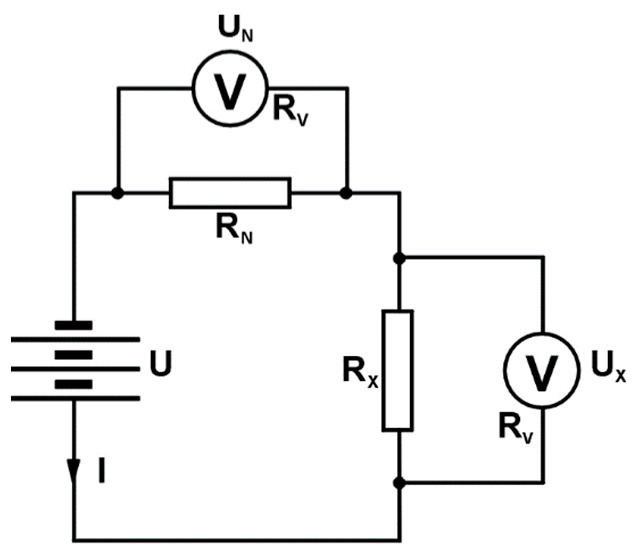
Scheme of the series measuring circuit.

According to this method, a resistor of known resistance R_N_ connects to the voltage of the source U in series (Figure 1), or with a resistor of unknown resistance R_X_. The value of the unknown resistance is obtained from the value of the known resistance and the ratio of voltage drops—i.e., currents—on both resistors [35].

In the case of a series connection with resistors, the voltages are measured with an internal resistance voltmeter R_V_. With this connection, the voltage drops across the resistors R_X_ and R_N_ are:

(1) $$U_{X}=I\cdot \frac{R_{X}\cdot R_{V}}{R_{X}+R_{V}};{\;U}_{N}=I\cdot \frac{R_{N}\cdot R_{V}}{R_{N}+R_{V}}$$

If the resistances R_X_ and R_N_ are significantly less than R_V_, the impact of R_V_ can be neglected, from which follows the expression (2):

(2) $$R_{X}=R_{N}\cdot \frac{U_{X}}{U_{N}}$$

A series connection is used to measure low-value resistance. To measure smaller resistances (of the order of magnitude of several hundred ohms), a series measurement connection (Figure 1) is used, which was also used in this research.

### 2.2. Force–Elongation Diagram for the Fabric

**Figure 2:**
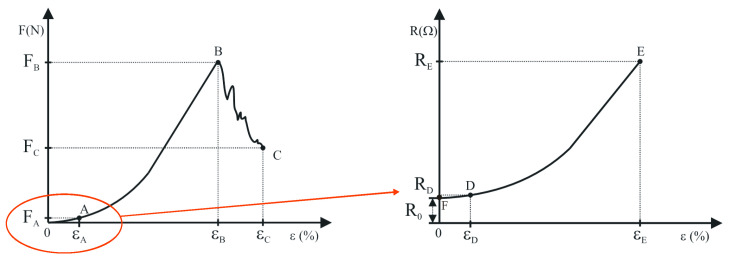
Characteristic diagrams of the tensile force–elongation (F-ε) curve of the woven fabric and the electrical resistance–elongation (R-ε) curve of the conductive fabric yarn.

The functional relationship between tensile force and elongation cannot be determined theoretically, but only by experimentally testing samples made of a specific material. Fabrics are a special type of anisotropic, inhomogeneous material [36,37]. In the biaxial structure of weaving, two main directions are defined: longitudinal (warp), and transverse (weft). Experiments determine the relationship between force and elongation in the form of diagram. Mechanical characteristics are investigated in the elastic range [38,39]. Under the action of tensile force F, elongation ε of the sample occurs. Figure 2 shows the force–elongation curve of the fabric.

The yarns in the fabric are arranged in a wavy shape. Due to the corrugation of the yarns, the fabric behavior in the elongation at the warp and the weft will consist of a range that mainly occurs due to the correction of the corrugation of the yarns and a range that results from the elongation of the yarns themselves. The final elongation at the break of the fabric will depend on the size of the corrugation, and on the possibility of this corrugation being straightened, which is determined by the pliability of the other yarn system.

The curve in the tensile force–elongation diagram (Figure 2) consists of two parts [40,41]: The first part 0A is a linear region representing the elastic area of the fabric, in which the yarns move into the fabric embroidery and the corrugation of the yarns in the fabric is corrected. Up to point A, the linear dependence of tensile force and fabric elongation applies. Thus, Hooke’s law of anisotropic material behavior can be applied to this region. The second part AB is nonlinear. As the force increases, elastoplastic deformation occurs. In this part, in addition to the movement of the yarns in the fabric, the yarns are unloaded in their elastic range, and the fabric sample is elongated due to the elongation of the sample itself. With a further increase in force, the fabric takes on plastic deformations. Individual yarns of the sample break, and then the sample breaks completely. Point B is the maximum elongation force.

In further considerations, the behavior of the fabric in the elastic range—i.e., up to point A—is observed. The elastic response of fabrics to tensile force is modelled using the idea that tensile force F is expressed in the region 0A as a linear function of elongation ε:

(3) F=k·ε 

where k is the elasticity coefficient of the material.

The polynomial function is applied for modelling the elastoplastic deformation, expressed as:

(4) $$F=Q\left(\varepsilon \right)=a_{n}\cdot \varepsilon^{n}+\cdots +a_{1}\cdot \varepsilon +a_{0}\;$$

where a_0_, a_1_, …, and a_n_ are known coefficients.

The least squares method is used for fitting part 0A and determining the coefficients k, a_0_, a_1_, …, and a_n_. Determining point A between the linear and nonlinear parts (Figure 2) represents the most sensitive part of the problem.

### 2.3. Mathematical Model of Force and Elongation Dependence

A mathematical model of the dependence of the force F and the elongation ε is set up, which can be expressed as:

(5) $$F\left(\varepsilon \right)=\{\begin{array}{ll} k\cdot \varepsilon , & 0\leq \varepsilon \leq \varepsilon_{A}\\ Q\left(\varepsilon \right), & \varepsilon_{A}\leq \varepsilon \leq \varepsilon_{B} \end{array}$$

At transition point A, the conditions of equality must be met as follows:

(6) $$k\cdot \varepsilon_{A}=Q\left(\varepsilon_{A}\right)\;$$

The curve F-ε (Figure 2) must be continuous—that is, the transition at point A must be smooth. Thus, the equality condition (Equation (6)) and the condition of matching the differentials of Equation (7) at point A must be satisfied:

(7) $$k=Q^{\prime }\left(\varepsilon_{A}\right)$$

The polynomial Q of the lowest degree that would meet both the above conditions of Equations (6) and (7) must have four coefficients, or the degree of the polynomial Q must be n = 3, expressed as:

(8) $$Q\left(\varepsilon \right)=a_{3}\cdot \varepsilon^{3}+a_{2}\cdot \varepsilon^{2}+a_{1}\cdot \varepsilon +a_{0}\;$$

The parameters of the function F (ε) shown in Equation (5) are estimated by the least squares method. Estimates of regression parameters are determined such that Equation (9) holds:

(9) $$\overset{N}{\underset{i=1}{\sum }}{\left(F\left(\varepsilon_{i}\right)-F_{i}\right)}^{2}\to \min$$

where ε_i_ and F_i_ are experimentally obtained values of elongation and associated force, respectively, while F is the theoretical value of force.

### 2.4. Electrical Resistance–Elongation Diagram for the Fabric, and Mathematical Model

When a tensile force acts on a fabric sample, the sample is stretched in the direction of the force, and its lateral constriction—which is perpendicular to the direction of the force. This results in a change in the electrical resistance and conductivity of the conductive yarns woven into the fabric. Based on the experimentally obtained values, a connection can be obtained between the electrical resistance of the conductive yarns and the corresponding relative elongation, as shown by the R-ε diagram in Figure 2.

The initial resistance of the conductive yarn is denoted by R_0_. The curve in the electrical resistance–elongation diagram (Figure 2) consists of a linear part AD and a nonlinear part located on the curve between points D and E. Point E represents the maximum value of electrical resistance R_E_ at elongation ε_E_. In the initial range of the diagram up to point D the electrical resistance is represented as a linear function of extension:

(10) R=p·ε+s 

where p is the coefficient of the slope of the line and s is section of the line.

A polynomial function can be applied to model the nonlinear part of the curve, expressed as:

(11) $$R=H\left(\varepsilon \right)=b_{n}\cdot \varepsilon^{n}+\cdots +b_{1}\cdot \varepsilon +b_{0}\;$$

where b_0_, b_1_, …, and b_n_ are known coefficients.

Determination of the coefficients p, b_0_, b_1_, …, and b_n_ is solved by the least squares method. A mathematical model of the dependence of the electrical resistance R and the extension ε is set, which can be written as:

(12) $$R\left(\varepsilon \right)=\{\begin{array}{c} p\cdot \varepsilon +s,\;\;0\leq \varepsilon \leq \varepsilon_{D}\\ H\left(\varepsilon \right),\;{\;\varepsilon }_{D}\leq \varepsilon \leq \varepsilon_{E}\;\; \end{array}\;$$

To determine the transition point D, the condition of equality must be met:

(13) $$p\cdot \varepsilon_{D}+s=H\left(\varepsilon_{D}\right)\;$$

The curve R-ε (Figure 2) must be continuous—i.e., the transition at point D must be smooth—and, therefore, the equality condition of Equation (13) must be satisfied, and the differentials of the functions at these points must coincide:

(14) $$p=H^{\prime }\left(\varepsilon_{D}\right)$$

The function that meets the conditions of Equations (13) and (14) is a polynomial B, which must be third-degree (n = 3):

(15) $$H\left(\varepsilon \right)=b_{3}\cdot \varepsilon^{3}+b_{2}\cdot \varepsilon^{2}+b_{1}\cdot \varepsilon +b_{0}\;$$

The parameters of the function R (ε) shown in Equation (12) can be estimated by the least squares method. Estimates of regression parameters can be determined such that Equation (16) holds:

(16) $$\overset{N}{\underset{i=1}{\sum }}{\left(R\left(\varepsilon_{i}\right)-R_{i}\right)}^{2}\to \min$$

where ε_i_ and R_i_ are experimentally obtained values of elongation and associated resistance, respectively, while R is the theoretical value of resistance.

## 3. Experimental Part

### 3.1. Samples of the Conductive Yarns

The conductive yarns used for this paper were purchased from Shieldex Trading Inc. (Bremen, Germany). The ECYs are silver-coated products that have antibacterial properties and are thermally and electrically conductive [42].

In the experimental part of the paper, three different ECYs are used (designations X, Y, and Z). All three ECYs are made of high-strength polyamide (PA 6.6), and are coated with 99% pure silver. The yarn with designation X is single and has 17 filaments; its yarn count after coating with silver is 142 dtex. The yarn with designation Y is double and has 34 filaments; its yarn count after silver plating is 295 dtex. The yarn with designation Z is double and has 72 filaments; its yarn count after silver plating is 604 dtex. The most important properties of these ECYs are shown in Table 1.

### 3.2. Fabrics Samples

Fabric samples were made in a particularly careful manner in a real industrial process on an air-jet loom, which was computer-controlled, with the required weaving parameters. In this way, the electrically conductive threads were woven into the textile material during the weaving process, in the same manner as they would be in the case of real production of the electrically conductive textiles, with controlled and repeatable parameters.

The experiments were performed on fabric samples a with structurally identical plain weave from a mixture of cotton (50%) and polyamide (50%) yarns, a warp yarn count of Tt = 33 × 2 tex, and a weft yarn count of Tt = 50 tex. Table 2 shows the data for the underlying fabric.

The yarn count of the yarn was determined via the gravimetric method, according to ISO 2060:1994. Fabric density was tested according to ISO 7211-2:1984 [43]. The thickness of the fabric was determined according to ISO 5084:1996 [44]. Prior to the testing, the fabrics with woven ECYs were conditioned for 24 h under normal conditions: 65 ± 2% humidity, at a temperature of 20 °C ± 2 °C. ECYs were woven into the fabric in the direction of the weft and warp yarns in the plain weave. ECYs are shown in red, and warp yarns in blue.

**Figure 3:**
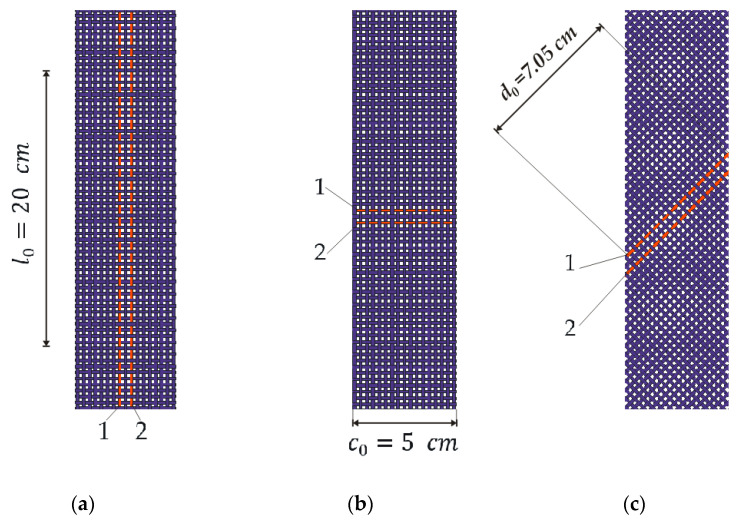
Fabric samples with woven ECYs: (**a**) sample cut in the weft direction (0°); (**b**) sample cut in the warp direction (90°); (**c**) sample cut at a 45° angle.

For the purposes of this experiment, three fabrics with different ECY yarn counts of (X, Y, and Z) were designed and woven. The samples of fabrics with ECYs were cut in three different directions: the direction of the weft (90°), where the length of the electrically conductive yarn was equal to the length of the sample, and amounted to l_0_ = 20 cm, (Figure 3a); the direction of the warp (0°), where the length of the conductive yarn was equal to the width of the sample, and amounted to c_0_ = 5 cm (Figure 3b); and at an angle of 45° to the weft, where the length of the conductive yarn d_0_ = c_0_·$\sqrt{2}=7.05\;\mathrm{cm}$ (Figure 3c). The direction of action of the tensile force during the performance of the experiment was always the same. For each specified cutting direction of the electrically conductive fabric sample, and for each yarn count, five measurements were performed.

In each cut fabric sample, there are two ECYs denoted with the numbers 1 and 2. Both ECYs in the sample were of equal yarn counts; the electrical resistance of yarn 1 is denoted by R_1_, and that of yarn 2 is denoted by R_2_.

### 3.3. Method and Manner of Measuring the Electrical Resistance of Electrically Conductive Yarns

For the purposes of this research, the following measuring equipment was used: a device for measuring the breaking force of the Textechno Statimat ME+ sample tensile strength tester, (Textechno H. Stein GmbH & Co. KG, Moenchengladbach, Nordrhein-Westfalen, Germany) which is fully automated, microprocessor-controlled, and works on the principle of constant strain rate; a measurement assembly (Faculty of Textile Technology, Zagreb, Croatia), purpose-designed via the resistance comparative method, an optoelectronic start signal adjustment assembly, an analog-to-digital converter (NI USB-6212),( National Instruments, Austin, TX, USA) and PCs (HP, Houston, TX, USA) to control the measurement process and collect the measured data. In addition to measurement, the PCs were used for the development of appropriate measurement and analytical software (LabVIEW 2019, NI, Austin, TX, USA), as well as for conducting a complete analysis of measured data, and for the presentation of results.

**Figure 4:**
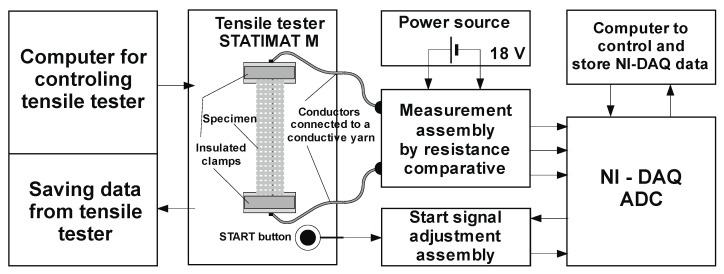
System for measuring changes in the electrical resistance of woven conductive yarns.

**Figure 5:**
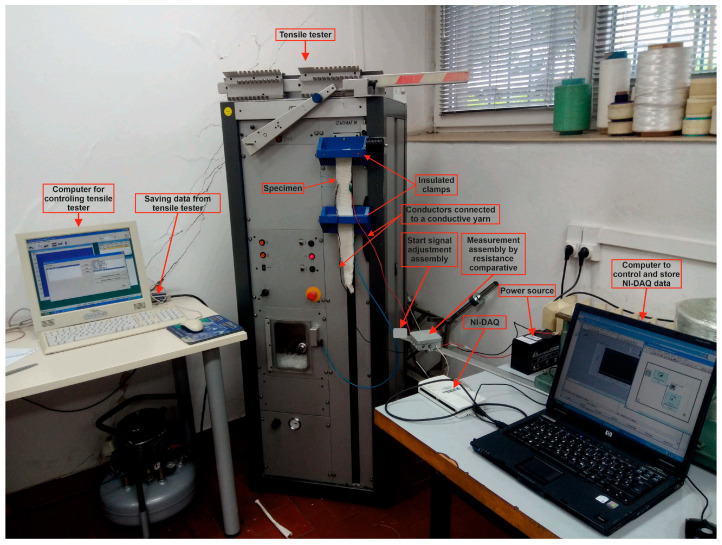
Photo of the measuring system.

To measure and collect the research results, a measuring system was designed and manufactured, as shown in concept in Figure 4, and realized in Figure 5.

In addition to the preparation and processing of samples, measurement and control software was used for the precise monitoring of changes in the resistance of the electrically conductive thread for every 0.02 mm of elongation. For this purpose, the values of the magnitude of the stress from the resistance-measuring assembly were read by a comparative method, and a change in the resistance value of the conductive filament was processed for each elongation value. The data were read at a speed of 50 measurements per second and stored for further processing.

The sample of standard dimensions was fixed to the galvanically insulated clamps of the tensile tester at a distance of l_0_ = 200 mm, and copper conductors for measuring voltage change—i.e., resistance—were connected to the ECYs by the process of fixing copper clips (crimping). The samples were subjected to uniaxial tensile loading at a tensile speed of v = 100 mm/min until a break was reached. The tensile properties of all samples were tested according to ISO 13934-1:2008 with a tensile tester.

The START button of the tensile tester is simultaneously connected to the ADC via the start signal adjustment assembly. This allows us, by pushing the START button, to simultaneously start measuring and storing the force at the break and the elongation data from the tensile tester, as well as the voltage signals from the sample, to calculate the resistance change.

## 4. Results and Discussion

**Figure 6:**
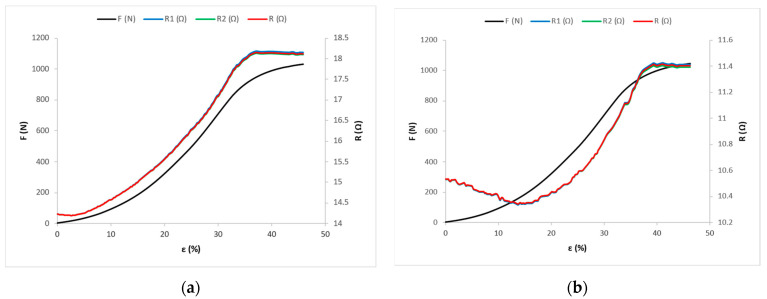

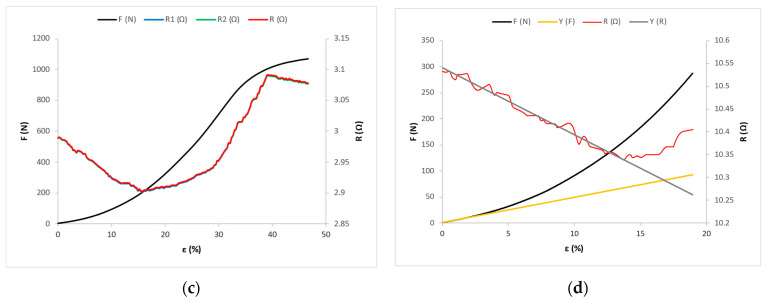
Force–elongation (F-ε) and electrical resistance–elongation (R-ε) diagrams for fabric samples cut in the warp direction: (**a**) for sample X-0; (**b**) for sample Y-0; and (**c**) for sample Z-0. (**d**) Experimental and mathematical models of F-ε and R-ε curves for sample Y-0.

**Figure 7:**
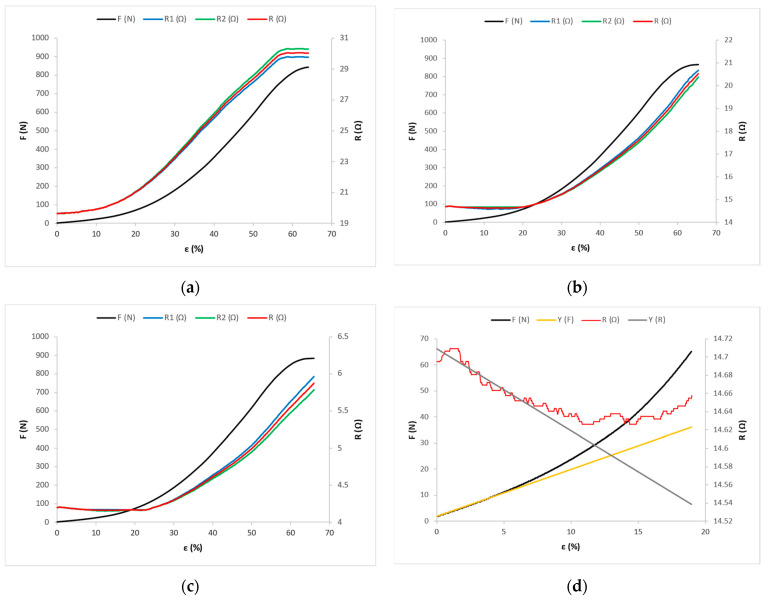
Force–elongation (F-ε) and electrical resistance–elongation (R-ε) diagrams for fabric samples cut at a 45° angle: (**a**) for sample X-45; (**b**) for sample Y-45; and (**c**) for sample Z-45. (**d**) Experimental and mathematical models of F-ε and R-ε curves for sample Y-45.

**Figure 8:**
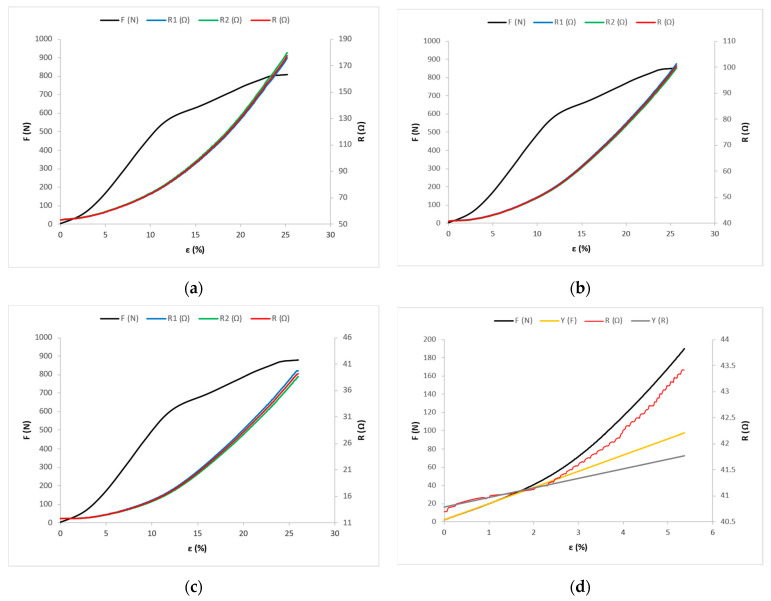
Force–elongation (F-ε) and electrical resistance–elongation (R-ε) diagrams for fabric samples cut in the weft direction: (**a**) for sample X-90; (**b**) for sample Y-90; and (**c**) for sample Z-90. (**d**) Experimental and mathematical models of F-ε and R-ε curves for sample Y-90.

Under the action of tensile force F, the corresponding longitudinal deformation is measured, i.e., the elongation ε of the sample and the electrical resistance of the conductive yarns R. Mean values of the results of measuring the action of tensile force F and associated elongation ε, and mean values of electric resistance R for the samples cut at 0°, 45°, and 90° angles, are shown in the Figure 6, Figure 7 and Figure 8.

X-0 is the designation of a fabric sample with a yarn count (X) cut at a 0° angle; X-45 is the designation of a fabric sample with a yarn count (X) cut at a 45° angle; X-90 is the designation of a fabric sample with a yarn count (X) cut at a 90° angle.

Y-0 is the designation of a fabric sample with a yarn count (Y) cut at a 0° angle; Y-45 is the designation of a fabric sample with a yarn count (Y) cut at a 45° angle; Y-90 is the designation of a fabric sample with a yarn count (Y) cut at a 90° angle.

Z-0 is the designation of a fabric sample with a yarn count (Z) cut at a 0° angle; Z-45 is the designation of a fabric sample with a yarn count (Z) cut at a 45° angle; Z-90 is the designation of a fabric sample with a yarn count (Z) cut at a 90° angle.

R_1_ denotes the mean value of the measured electrical resistance of the electrically conductive yarn 1; R_2_ denotes the mean value of the measured electrical resistance of the electrically conductive yarn 2 (Figure 3). The mean value of the electrical resistance of conductive yarns 1 and 2 is denoted by R = (R1 + R2)/2.

Figure 6a shows the distributions of tensile force and electrical resistance as functions of elongation for the fabric sample X-0, Figure 6b for the fabric sample Y-0, and Figure 6c for the fabric sample Z-0.

In the linear area, the slope of the line is important for the analysis—not its section on the ordinate—and, therefore, in further considerations, the section on the ordinate will not be shown or considered (Figure 6d, Figure 7d, and Figure 8d). The experimental force–elongation curves (F-ε) and electrical resistance–elongation curves (R-ε) are presented, as well as the corresponding analytical models in the first zone (linear) predicting the force–elongation curve and the electrical resistance–elongation curve for the fabric samples: Y-0 (Figure 6d); Y-45 (Figure 7d); and Y-90 (Figure 8d). Figure 6d shows the representation only for sample Y-0, and the representations for X-0 and Z-0 are very similar and are made in a similar way, so they are not shown here. The same reasoning applies to the representations in Figure 7d and Figure 8d.

Figure 7a shows the tensile force and electrical resistance distributions as functions of the X-45 fabric sample, Figure 7b shows the Y-45 fabric sample, and Figure 7c shows the Z-45 fabric sample.

Figure 8a shows the tensile force and electrical resistance distributions as functions of the X-90 fabric sample, Figure 8b shows the Y-90 fabric sample, and Figure 8c shows the Z-90 fabric sample.

The mean values for the experimentally obtained maximum force F_B_ and the corresponding elongation ε_B_, along with the coefficients of variation CV, are shown in Table S1.

The maximum force F_B_ has the highest value for sample Z-0 with the highest yarn count cut in the warp direction—amounting to F_B_ = 1068.8 N—and the lowest value for sample X-90 with the lowest yarn count, cut in the weft direction, amounting to F_B_ = 809.1 N. The maximum elongation ε_B_ has the highest value for sample Z-45—amounting to ε_B_ = 65.98%—and the lowest value for sample X-90, amounting to ε_B_ = 25.22%. For samples cut in the same direction, the value of maximum force and elongation increases with the increase in the yarn count of the electrically conductive yarn.

The mean values of the measured initial electrical resistance of the electrically conductive yarn 1 shown in Table S2 are denoted by R_01_. The mean values of the initial electrical resistance of the electrically conductive yarn 2 are denoted by R_02_, and the mean value of the initial resistance of the conductive yarns 1 and 2 is R_0_ = (R_01_ + R_02_)/2. The mean values of the measured maximum electrical resistance at point E of the electrically conductive yarn 1 are denoted by R_E1_. The mean values of the measured maximum electrical resistance of the electrically conductive yarn 2 are denoted by R_E2_, and the mean value of the maximum electrical resistance of the electrically conductive yarns 1 and 2 is R_E_ = (R_E1_ + R_E2_)/2.

In samples cut in the same direction, the mean values of the initial electrical resistance and the maximum electrical resistance decrease with the increase in the yarn count of the electrically conductive yarn. The maximum electrical resistance R_E_ has the highest value for sample X-90 with the lowest yarn count—which is cut in the weft direction and amounts to R_E_ = 177.69 Ω—and has the lowest value for sample Z-0 with the highest yarn count, which is cut in the warp direction and amounts to R_E_ = 3.09 Ω. The initial electrical resistance has the highest value for sample X-90— amounting to R_0_ = 53.40 Ω—and the lowest value for sample Z-0, amounting to R_0_ = 2.99 Ω.

In further considerations, the change in tensile force and electrical resistance, along with the behavior of the fabric with ECYs, will be observed in the linear region alone. The values of point A at which the force–elongation curve passes from the elastic range—which is linear—to the nonlinear range are shown by the coordinates (ε_A_, F_A_) given in Table S3 for different samples of fabrics with ECYs. Point A is common to the experimental curve and the mathematical model; it is obtained from Equations (6) and (7).

For the corresponding mathematical models, the corresponding coefficients of the linear function were calculated, i.e., the lines k (range OA) and the coefficients a_0_, a_1_, a_2_, and a_3_ of the cubic parabola (nonlinear range). The values of these coefficients were calculated using Equations (4), (5) and (8), and are shown in Table S3. The correlation coefficients r between the experimental curve and the mathematical model were calculated, and are shown in Table S3. The correlation coefficients show very high congruence of the experimental curves and the mathematical model in the linear and nonlinear ranges. The equation of lines and of the third-order polynomial describe very well the curve of the ratio between the experimentally obtained values of the forces and the corresponding elongations.

For samples cut in the same direction, the values of force and elongation at point A increase with the increase in the yarn count of the conductive yarn. Furthermore, in the linear part of the diagram, the slope of the line increases with these samples. For fabric samples cut in the weft direction (X-90, Y-90, and Z-90) the slopes of the lines in the linear range are the highest, and for samples cut at an angle of 45° the slopes of the lines are the lowest. Therefore, the samples X-90, Y-90, and Z-90 have the highest values of force and the lowest values of elongation at point A.

The values of point D at which the electrical resistance–elongation curve passes from an elastic range that is linear to a nonlinear range are shown by the coordinates (ε_D_, F_D_) given in Table S4 for different samples of fabric with ECYs. Point D is common to the experimental curve and the mathematical model, and is obtained from Equations (13) and (14).

For the corresponding mathematical models, the associated coefficients of the linear function—i.e., the lines p (range OD) and the coefficients b_0_, b_1_, b_2_, and b_3_ of the cubic parabola (nonlinear range)—were calculated. The values of these coefficients were calculated using Equations (11), (12) and (15), and are shown in Table S4. The associated correlation coefficients r between the experimental curve and the mathematical model were also calculated, and are given in Table S4. The correlation coefficients show good congruence of the experimental curves and the mathematical model in the linear and nonlinear ranges. The equation of lines and the third-order polynomial describe well the curve of the ratio of the experimentally measured values of electrical resistance and the corresponding elongations.

For samples cut in the same direction, the electrical resistance values decrease and the elongations at point D increase with the increase in the yarn count of the conductive yarn. For fabric samples cut in the warp direction (X-0, Y-0, and Z-0) and at a 45° angle (X-45, Y-45, and Z-45), in the OD range, the line direction coefficient p has a negative value (Table S4), so the values of electrical resistance decrease from the initial value of resistance R_0_ to point D, where they have the value R_D_ (Figure 6a–c and Figure 7a–c). For these samples, in the linear part of the force–elongation dependence diagram, the direction coefficient of line k has a positive value (Table S3, Figure 6a–c and Figure 7a–c). Thus, in the linear part, the values of tensile forces and elongation increase, and at the same time the values of electrical resistance decrease (Tables S3 and S4). It can be freely assumed that in the structure of the electrically conductive yarn there is an increase in the number of parallel contact resistances between the filaments, with a simultaneous decrease in series resistances on the surface, which causes an overall decrease in electrical resistance at the tested length of the electrically conductive yarn.

For fabric samples cut in the weft direction (X-90, Y-90, and Z-90), in the OD range, the direction coefficient of line p has a positive value (Table S4)—i.e., the electrical resistance values increase from the initial resistance value R_0_ towards point D, where they have the value R_D_ (Figure 8a–c). For these samples, in the linear part of the force–elongation diagram, the direction coefficient of line k also has a positive value (Table S3), and the values of the tensile forces and elongation increase, as do the values of electrical resistance (Tables S3 and S4). In these samples, there is a direct action of force and elongation on the conductive yarns, which are consequently elongated, reducing their waviness, which increases their electrical resistance. Due to the action of force and tension, there is an increased number of surface series resistances with simultaneous interruptions of contacts in the structure (between filaments), which eliminates parallel joints; thus, there is a noticeable increase in total electrical resistance on the tested length of woven electrically conductive yarn.

**Figure 9:**
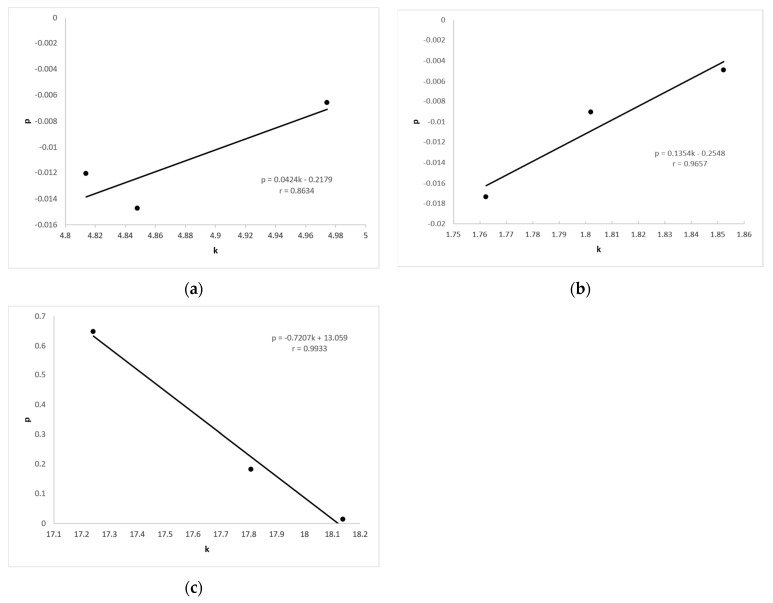
Correlation between the slope of the line k of force and the slope of the line p of electric resistance for samples: (**a**) X-0, Y-0, and Z-0; (**b**) X-45, Y-45, and Z-45; and (**c**) X-90, Y-90, and Z-90.

Figure 9 shows the correlation between the slope of the line k, showing the dependence of force and elongation, and the slope of the line p, showing the dependence of electrical resistance and elongation. High correlation coefficients r were obtained, and the highest were for samples X-90, Y-90, and Z-90, amounting to r = 0.9933.

If the values of electrical resistance at point D (R_D_) are compared (Table S4) for samples of the same yarn count that are cut in different directions (X-0, X-45, and X-90), (Y-0, Y-45, and Y-90), and (Z-0, Z-45, and Z-90), it can be concluded that the values of electrical resistance are the lowest when the samples are cut in the warp direction, and the highest for the samples cut in the weft direction (14.19 Ω, 19.53 Ω, and 54.87 Ω), (10.34 Ω, 14.64 Ω, and 41.20 Ω), and (2.92 Ω, 4.17 Ω, and 11.81 Ω), respectively. Samples cut in the direction of the warp (X-0, Y-0, and Z-0) have a 5-cm length of electrically conductive yarn, which is located at the sample perpendicular to the direction of the tensile force. Under the action of force, the sample is elongated in the direction of the force, and in the transverse direction (perpendicular to the direction of force action) there is a lateral narrowing of the sample and an increase in the corrugation of the electrically conductive yarn. In doing so, its cross-section increases on parts of the yarn due to compression, which has the effect of reducing the electrical resistance. Here we should also assume surface interruptions of series-connected elements, with a simultaneous significant increase in the number of parallel joints in the yarn structure, so it is logical that the total resistance at the measured yarn length decreases.

Samples cut at a 45° angle (X-45, Y-45, and Z-45) have an electrically conductive yarn length of 7.05 cm. In these samples, the length of the electrically conductive yarn is greater than in the sample cut in the direction of the warp, so this assumes a higher value of the initial electrical resistance R_0_.

The samples cut in the weft direction (X-90, Y-90, and Z-90) have an electrically conductive yarn length of 20 cm, which is placed in the sample parallel to the direction of action of the tensile force. Under the action of force, the sample and the length of the electrically conductive yarn extend in the direction of the force, which leads to a large increase in electrical resistance. As the length of the electrically conductive yarn is proportional to the value of the electrical resistance, it has been proven that the value of electrical resistance is affected by yarn count, but also by the change in the lengths of the electrically conductive yarn in the sample in relation to the direction of action of the tensile force.

## 5. Conclusions

The results of the experiment conducted in this paper indicate that the electrical resistance of the woven ECY increases or decreases depending on the strength of the force as well as the direction of force (i.e., elongation of the fabric). This effect can be characterized from a negative or a positive aspect. If fabrics with woven ECY are used as energy conductors, then in the event of elongation the resistance changes, so part of the conducted energy is converted into joule heat, and the supply voltage also changes—which is unfavorable. Likewise, if a fabric with woven ECY is used as a conductor for the transmission of measurement signals, then as a result of the elongation of the material, a measurement error may occur due to a change in resistance.

The positive side of the change in the resistance of woven yarns may be for the production of textile sensors for registering changes in the state of textile materials by measuring force, i.e., elongation.

Therefore, the examination of the impact of elongation on the change in the electrical resistance of the ECYs woven into the fabric is very significant. The aim of this paper is to examine how the change in the elongation of the samples under the action of tensile force affects the change in the electrical resistance of the ECYs woven into fabrics.

The impact of the action of tensile force in different directions on the fabric with woven ECY was examined. Elongation of the fabric results in a change in the electrical resistance of the woven electrically conductive yarn. Based on the experimental results, we obtained the ratio curves of the magnitudes of tensile forces and associated elongations, as well as the curves of the ratios of electrical resistance and elongation values, for directions when the force acts in the warp direction, in the weft direction, and at an angle of 45°.

A mathematical analysis was performed by which the appropriate analytical model was connected to the experimental curves of force–elongation and electrical resistance–elongation. The analytical model describing the curves of force–elongation and electrical resistance–elongation has two ranges: linear and nonlinear.

The associated correlation coefficients r between the experimental curve and the mathematical model were calculated, and showed a very high correspondence of the experimental curve and the mathematical model in the linear and nonlinear ranges. The correlation coefficient r is between 0.9945 and 0.9999 for the force–elongation curve, and between 0.9813 and 0.9999 for the electrical resistance–elongation curve.

Research into the impact of the action of force on the change in the electrical resistance of an electrically conductive yarn in a fabric was conducted in a linear elastic range. The following conclusions can be drawn from the conducted research and the analysis of the results:

For samples cut in the same direction, the values of tensile force and elongation increase, while the values of electrical resistance decrease, with the increase in the yarn count of the electrically conductive yarn.

The value of electrical resistance is impacted by the yarn count, but also by the length of the electrically conductive yarn in the sample in relation to the direction of action of the tensile force. As the length of the electrically conductive yarn increases and its cross-section decreases, the electrical resistance increases. It can be freely assumed that the change in the total resistance is also impacted by changes in series-parallel elements in the structure and surface of the electrically conductive threads when they are exposed to the action of elongation force.

This research provides information and conclusions about the influence of tensile force on changes in the electrical resistance of conductive threads woven into fabrics. The obtained results will be used for future research aimed at developing a stretchable and sensitive strain sensor for smart textiles and clothing.

Based on the conducted measurements and experimentation for the purpose of incorporating woven sensors into e-textiles—smart and intelligent clothing—the textile ribbon with ECYs was made using the traditional hand-weaving technique. Such sensors are integrated into smart clothing for apnea patients and infant clothing for the purpose of monitoring breathing as textile-embedded interconnects [32], recording the change in resistance, which is converted into voltage signals, leading to the input of electronic circuits for breathing detection, i.e., chest movements. The realized prototype of this type of clothing has been awarded a number of prizes at world innovation exhibitions, which is an indicator of the innovativeness of the approach [45]. The presented sensor had the simple task of detecting respiratory arrest, where the accuracy of the sensor and its hysteresis were not of primary importance, as with most of the sensors presented in the introductory part of this paper. The main advantages of the presented sensor are its reliability, simplicity of construction, suitability for conventional textile production processes, and pleasant textile feel.

Woven textile sensors with ECYs have proven to be excellent for incorporation into clothing, given that they are predominantly textile materials that are comfortable interacting with the skin of the human body.

## Figures and Tables

**Table 1:** Characteristics parameters of the ECYs.

Code Name	X	Y	Z
Material	Polyamide 6.6 filament	Polyamide 6.6 filament	Polyamide 6.6 filament
Metal-plated	99% Pure silver	99% Pure silver	99% Pure silver
Coating	Yes	Yes	Yes
Filaments	17	17	36
Ply	1	2	2
Yarn count, raw (dtex)	117f17	117f17	235f36
Yarn count, silverized (dtex)	142	295	604
Resistivity	<500 Ω/m	<300 Ω/m	80 Ω/m

**Table 2:** Test results for the basic fabric parameters.

	Warp Direction			Weft Direction				
Fabric Structure	Yarn Fibers	Yarn Count (tex)	Density (cm^−1^)	Yarn Fibers	Yarn Count (tex)	Density (cm^−1^)	Weight (g/m^2^)	Fabric Thickness (mm)
Plain weave	50%Cotton/ 50%PA	33 × 2	33	50%Cotton/ 50%PA	50	25	208.2	0.463

## Data Availability

Not applicable.

## Supplementary Materials

The following are available online at https://www.mdpi.com/article/10.3390/ma14123390/s1, Table S1: The mean values of maximum force FB, the corresponding elongation εB, and the coefficients of variation CV; Table S2: The mean values of initial R0 and maximum electrical resistance RE, and respective variation coefficients; Table S3: The coordinates of point A, coefficients k, a0, a1, a2, and a3 of the curves of the mathematical model, and correlation coefficient r; Table S4: The coordinates of point D, coefficients p, b0, b1, b2, and b3 of the curves of the mathematical model, and correlation coefficient r.
