# Supplementary material for: The Impact of Elongation on Change in Electrical Resistance of Electrically Conductive Yarns Woven into Fabric
Source: Materials (Basel). 2021 Jun 18;14(12):3390. doi: 10.3390/ma14123390 (PMC8235346; doi:10.3390/ma14123390)
Supplement: Supplementary file 1 [file materials-14-03390-s001.zip › materials-1226520-supplementary.pdf]

Supplementary materials

The Impact of Elongation on Change in Electrical Resistance of Electrically Conductive Yarns Woven into Fabric

Željko Knezić [1], Željko Penava [1], Diana Šimić Penava [2] and Dubravko Rogale [3,*]

[1] Department of Textile Design and Management, Faculty of Textile Technology, University of Zagreb, 10000 Zagreb, Croatia; zeljko.knezic@ttf.unizg.hr (Ž.K.); zpenava@ttf.unizg.hr (Ž.P.)
[2] Department of Engineering Mechanics, Faculty of Civil Engineering, University of Zagreb, 10000 Zagreb, Croatia; dianas@grad.unizg.hr
[3] Department of Clothing Technology, Faculty of Textile Technology, University of Zagreb, 10000 Zagreb, Croatia
* Correspondence: dubravko.rogale@ttf.unizg.hr

Table S1. The mean values of maximum force F_B and the corresponding elongation ε_B, and the coefficients of variation CV.

Fabric Tag	ε_B (%)		F_B (N)	
	$\bar{x}$ (%)	CV (%)	$\bar{x}$ (%)	CV (%)
**X-0**	45.80	8.64	1031.1	8.58
**Y-0**	46.24	7.95	1045.4	8.02
**Z-0**	46.70	8.12	1068.8	8.15
**X-45**	64.04	7.32	843.8	7.42
**Y-45**	65.36	8.18	865.8	8.11
**Z-45**	65.98	8.46	884.2	8.32
**X-90**	25.22	7.46	809.1	7.65
**Y-90**	25.72	7.82	853.7	7.81
**Z-90**	26.02	7.65	880.6	7.93

Table S2. Mean values of initial R_0 and maximum electrical resistance R_E, and variation coefficients.

Fabric	R_{01} (Ω)		R_{02} (Ω)		R_{E1} (Ω)		R_{E2} (Ω)		R_0 (Ω)	R_E (Ω)
Tag	$\bar{x}$ (Ω)	CV (%)	$\bar{x}$ (Ω)	CV (%)	$\bar{x}$ (Ω)	CV (%)	$\bar{x}$ (Ω)	CV (%)	$\bar{x}$ (Ω)	$\bar{x}$ (Ω)
**X-0**	14.21	1.50	14.25	1.43	18.19	2.99	18.13	2.84	14.23	18.16
**Y-0**	10.55	1.46	10.52	1.29	11.42	2.87	11.40	2.78	10.53	11.41
**Z-0**	2.98	1.28	3.00	1.32	3.09	2.74	3.09	2.97	2.99	3.09
**X-45**	19.61	1.31	19.66	1.37	29.79	2.68	30.32	2.61	19.64	30.06
**Y-45**	14.67	1.42	14.72	1.41	20.67	2.61	20.37	2.71	14.70	20.52
**Z-45**	4.19	1.48	4.21	1.49	5.97	3.01	5.79	3.05	4.20	5.88
**X-90**	53.38	1.37	53.42	1.51	175.72	2.71	179.66	2.69	53.40	177.69
**Y-90**	40.71	1.34	40.68	1.33	101.34	2.83	99.65	2.71	40.70	100.50
**Z-90**	11.77	1.44	11.73	1.46	39.74	2.91	38.60	2.85	11.75	39.17

Table S3. Coordinates of point A and coefficients of the curves of the mathematical model k, a_0, a_1, a_2, a_3 and correlation coefficient r.

Fabric	A		Linear Coefficient		Nonlinear Coefficients				
Tag	ε_A (%)	F_A (N)	k	r	a_0	a_1	a_2	a_3	r
**X-0**	1.38	8.41	4.81	0.9969	23.75	−13.91	2.062	−0.028	0.9960
**Y-0**	1.56	8.88	4.85	0.9945	26.50	−14.46	2.072	−0.028	0.9961
**Z-0**	1.74	10.40	4.97	0.9976	30.48	−15.10	2.096	−0.028	0.9962
**X-45**	3.78	8.49	1.76	0.9968	37.10	−9.94	0.644	−0.005	0.9938
**Y-45**	3.88	8.82	1.80	0.9989	43.48	−11.67	0.727	−0.005	0.9924
**Z-45**	3.96	9.15	1.85	0.9975	47.46	−12.65	0.775	−0.006	0.9918
**X-90**	1.12	21.19	17.24	0.9997	−34.47	49.69	0.036	−0.027	0.9883
**Y-90**	1.14	22.21	17.81	0.9998	−36.38	51.39	0.035	−0.027	0.9883
**Z-90**	1.16	22.96	18.14	0.9999	−37.91	52.47	0.030	−0.027	0.9883

Table S4. Coordinates of point D and coefficients of the curves of the mathematical model p, b_0, b_1, b_2, b_3 and correlation coefficient r.

Fabric	D		Linear Coefficient		Nonlinear Coefficients				
Tag	ε_D (%)	R_D (Ω)	p	r	b_0	b_1	b_2	b_3	r
**X-0**	2.78	14.19	−0.012	0.9985	14.21	−0.020	0.0059	−0.0001	0.9962
**Y-0**	13.82	10.34	−0.015	0.9813	11.61	−0.185	0.0079	−0.0001	0.9886
**Z-0**	14.96	2.89	−0.007	0.9836	2.98	−0.007	0.0001	0.0000	0.9937
**X-45**	6.06	19.53	−0.017	0.9982	19.77	−0.096	0.0098	−0.0001	0.9990
**Y-45**	7.64	14.64	−0.009	0.9984	14.98	−0.065	0.0026	0.0000	0.9996
**Z-45**	7.82	4.17	−0.005	0.9980	4.30	−0.024	0.0009	0.0000	0.9991
**X-90**	2.18	54.87	0.650	0.9983	53.23	0.436	0.1426	0.0014	0.9999
**Y-90**	2.24	41.20	0.184	0.9982	41.10	−0.215	0.1164	−0.0007	0.9999
**Z-90**	2.34	11.81	0.016	0.9978	12.13	−0.284	0.0637	−0.0005	0.9997
